# Hypoglycemia induces vascular endothelial dysfunction in subjects with normal glucose tolerance

**DOI:** 10.1038/s41598-022-06450-x

**Published:** 2022-02-16

**Authors:** Kenichi Tanaka, Yosuke Okada, Keiichi Torimoto, Kosuke Nishio, Manabu Narisawa, Yoshiya Tanaka

**Affiliations:** First Department of Internal Medicine, School of Medicine, University of Occupational and Environmental Health Japan, 1-1 Iseigaoka, Yahatanishi-ku, Kitakyushu, 807-8555 Japan

**Keywords:** Endocrinology, Endocrine system and metabolic diseases

## Abstract

This prospective study determined the effects of hypoglycemic stimulation on vascular endothelial function in non-diabetic patients using reactive hyperemia peripheral arterial tonometry (RH-PAT). The study included non-diabetic patients who were hospitalized for an insulin tolerance test (ITT) for the diagnosis of hypoadrenocorticism or hypopituitarism. Vascular endothelial function was assessed using the reactive hyperemia index (RHI) measured by the RH-PAT. We also measured the levels of anterior pituitary hormone, adrenaline, noradrenaline, and dopamine at the time of hypoglycemia. The primary endpoint was a change in the RHI at 120 min after insulin administration. The study included 27 patients. ITT was associated with significant increases in systolic blood pressure, pulse rate, and the blood levels of adrenocorticotropic hormone, cortisol, growth hormone, adrenaline, noradrenaline, and dopamine. RHI significantly decreased after ITT from 2.24 ± 0.51 to 1.71 ± 0.42. A significant inverse correlation was observed between the change in RHI and change in adrenaline (r = − 0.670, p = 0.012). We concluded that hypoglycemic stimulation altered vascular endothelial function, as measured by RH-PAT, even in patients free of glucose intolerance. The observed deterioration in vascular endothelial function correlated with increases in catecholamine levels during hypoglycemia.

Trial registration: UMIN000033244.

## Introduction

The pathological processes of vascular endothelial dysfunction and atherosclerosis increase the incidence of atherothrombotic events in patients with type 2 diabetes mellitus^1^. Furthermore, vascular endothelial dysfunction is an independent risk factor for the development of cardiovascular events^2,3^; and it can be induced by various metabolic abnormalities, such as obesity^4^, hypertension^5^, dyslipidemia^6^, and abnormal glucose metabolism^7^. Abnormal glucose metabolism pathology affects vascular endothelial dysfunction primarily through endothelial and smooth muscle dysfunction^8^, oxidative stress^9^, hyperinsulinemia associated with insulin resistance^10^, and hypoglycemia^11^. In particular, recent studies in patients with type 2 diabetes have reported that hypoglycemia is an independent risk factor for macrovascular events^12^. A meta-analysis study also showed that severe hypoglycemic episodes are associated with increased risk of cardiovascular disease in patients with type 2 diabetes^13^.

Using a cross-sectional study design, we reported previously that hypoglycemia was associated with vascular endothelial dysfunction in patients with type 2 diabetes^14^. Hyperglycemia, fluctuations in glucose levels^14^, and hypoinsulinemia^15^ were also found to affect vascular endothelial function in patients with type 2 diabetes mellitus. To assess whether hypoglycemia is directly related to vascular endothelial dysfunction, examining healthy individuals in whom the effects of hyperglycemia and fluctuations in glucose levels can be excluded is ideal. However, only a few studies have examined the effects of hypoglycemia on vascular endothelial function in healthy individuals^16,17^. It is also assumed that enhancement of the neuroendocrine and sympathetic nervous systems (increases in blood levels of cortisol, epinephrine, and norepinephrine^18^) during hypoglycemia can worsen vascular endothelial dysfunction^19^, however, there are no studies directly examining these relationships, and the pathophysiological mechanisms remain elusive.

The objectivity and reproducibility of the new device EndoPAT 2000 based on reactive hyperemia peripheral arterial tonometry (RH-PAT) has been recently established, and the device is currently used for non-invasive assessment of vascular endothelial function^20^. In this regard, the reactive hyperemia index (RHI) obtained by RH-PAT is considered useful in predicting cardiovascular diseases^21^.

**Figure 1:**
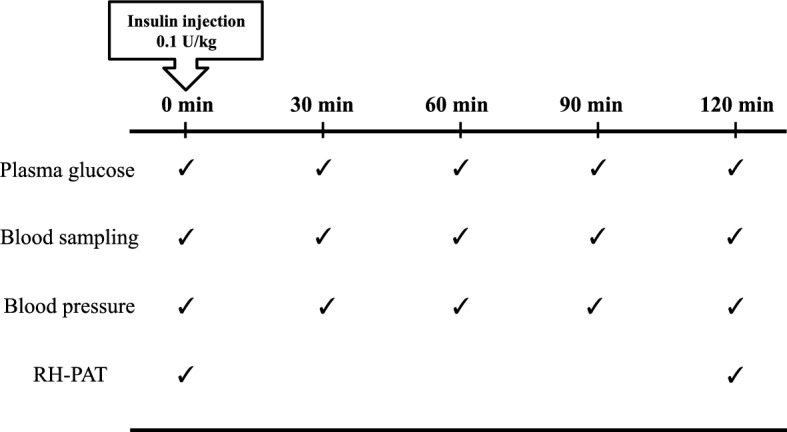
Study protocol. RH-PAT, reactive hyperemia peripheral arterial tonometry.

The present study was designed to determine the direct effect of hypoglycemia on vascular endothelial function in subjects free of abnormalities of glucose metabolism. Specifically, we determined the effect of a single hypoglycemic episode on vascular endothelial function in these individuals, as well as how this effect was related to the neuroendocrine and sympathetic nervous systems (Fig. 1).

## Results

### Clinical characteristics

Table 1 summarizes the background of the participating patients. The study included 27 patients (11 men), with a mean age of 51.4 ± 19.7 years; body mass index 23.5 ± 4.8 kg/m^2^, HbA1c 5.6 ± 0.3%, and fasting plasma glucose (FPG) 86.4 ± 7.5 mg/dL. The background conditions in these patients were anterior pituitary hyposecretion (of ≥ 2 pituitary hormones, n = 7), secondary adrenal insufficiency (n = 8), primary adrenal insufficiency (n = 2), latent adrenal insufficiency (n = 4), and growth hormone deficiency (n = 2), whereas 4 patients had normal pituitary function.

**Table 1:** Baseline clinical characteristics of the study participants.

	Participants (n = 27)
Male/female (n)	11/16
Age (years)	51.4 ± 19.7
BMI (kg/m^2^)	23.5 ± 4.8
sBP (mmHg)	117.9 ± 16.3
dBP (mmHg)	69.4 ± 16.0
HR (bpm)	63.9 ± 9.3
AST (IU/L)	26.3 ± 13.6
ALT (IU/L)	27.6 ± 34.8
GGT (IU/L)	42.5 ± 66.0
Cre (mg/dL)	0.73 ± 0.21
eGFR (mL/min/1.73m^2^)	80.4 ± 25.5
TG (mg/dL)	140.5 ± 99.1
HDL-C (mg/dL)	57.2 ± 23.4
LDL-C (mg/dL)	118.7 ± 33.5
FPG (mg/dL)	86.4 ± 7.5
HbA1c (%)	5.6 ± 0.3
ACTH (pg/mL)	23.0 ± 16.1
Cortisol (μg/dL)	6.9 ± 3.4
GH (ng/mL)	0.66 ± 1.47
IGF-I (ng/mL)	106.2 ± 69.5
Adrenaline (pg/mL)	27.2 ± 12.8
Noradrenaline (pg/mL)	269.0 ± 70.6
Dopamine (pg/mL)	9.6 ± 1.9
RHI	2.24 ± 0.51
Hypertension (%)	11(40.7)
Dyslipidemia (%)	13(48.1)
Antihypertensive agents (%)	10(37.0)
Antilipidemic agents (%)	4(14.8)
Smoking status (never/former/current; %)	17/6/4 (63.0/22.2/14.8)

Data are mean ± standard deviation, or n (%).

BMI, body mass index; sBP, systolic blood pressure; dBP, diastolic blood pressure; HR, heart rate; AST, aspartate transaminase; ALT, alanine transaminase; GGT, gamma-glutamyl transferase; Cre, creatinine; eGFR, estimated glomerular filtration rate; TG, triglyceride; HDL-C, high-density lipoprotein cholesterol; LDL-C, low-density lipoprotein cholesterol; FPG, fasting plasma glucose; HbA1c, glycated hemoglobin, GH, growth hormone; IGF-1, insulin-like growth factor-1; ACTH, adrenocorticotropic hormone; RHI, reactive hyperemia peripheral arterial tonometry index.

### Changes in cardiovascular responses, hormones and endothelial function on insulin tolerance test

**Figure 2:**
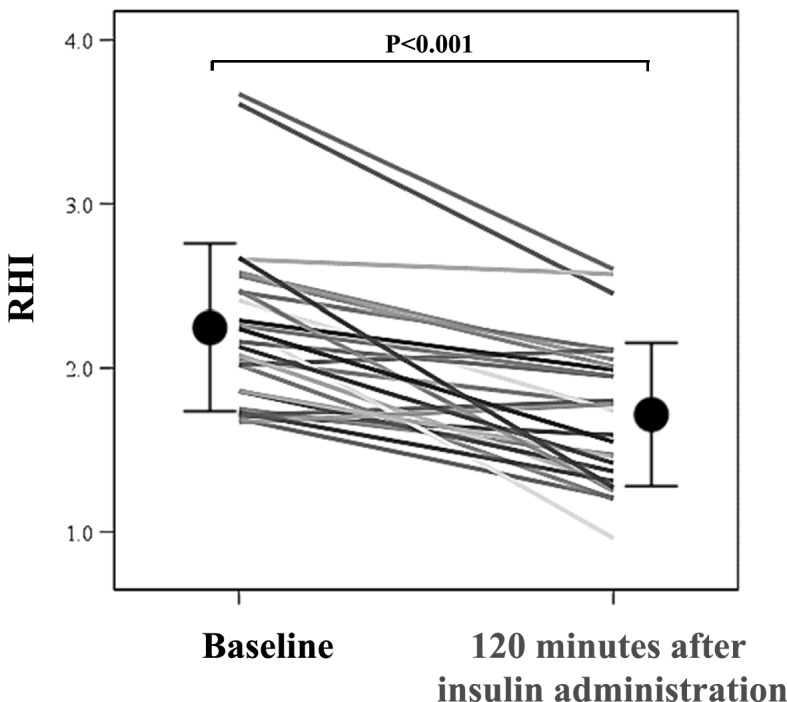
Changes in RHI during the insulin tolerance test. Circles: mean ± standard deviation. *P* < 0.001 vs. baseline, by Wilcoxon signed-rank test. RHI, reactive hyperemia index.

Table 2 shows the values of several parameters before and after the insulin tolerance test (ITT). Insulin administration induced hypoglycemia, with a mean lowest blood glucose value of 34.2 ± 8.6 mg/dL (range 20–50 mg/dL). None of the patients developed clinical signs of severe hypoglycemia (convulsion or loss of consciousness) during or after the ITT. Systolic blood pressure and heart rate (HR) increased significantly after the ITT. Similarly, significant increases were noted in blood levels of ACTH, cortisol, GH, adrenaline, noradrenaline, and dopamine. The RHI decreased significantly from 2.24 ± 0.51 before the load to 1.71 ± 0.42 after the load (Fig. 2). RHI values < 1.67 after loading were recorded in 13 (48.1%) patients.

**Table 2:** Insulin tolerance test.

	Baseline	Hypoglycemia^†^	*P*
sBP (mmHg)	117.9 ± 16.3	123.3 ± 17.4	0.009
dBP (mmHg)	69.4 ± 16.0	71.1 ± 11.4	0.227
HR (bpm)	63.9 ± 9.3	67.0 ± 9.8	0.002
PG (mg/dL)	86.4 ± 7.5	33.6 ± 9.5	< 0.001
ACTH (pg/mL)	23.0 ± 16.1	103.3 ± 70.9	< 0.001
Cortisol (μg/dL)	6.9 ± 3.4	13.8 ± 6.2	< 0.001
GH (ng/mL)	0.66 ± 1.46	7.5 ± 9.4	< 0.001
Adrenaline (pg/mL)	27.2 ± 12.8	524.4 ± 345.7	0.001
Noradrenaline (pg/mL)	269.0 ± 70.6	530.9 ± 339.0	0.001
dopamine (pg/mL)	9.6 ± 1.9	13.2 ± 2.6	0.003
RHI	2.24 ± 0.51	1.71 ± 0.44	< 0.001

Data are mean ± standard deviation. P values by Wilcoxon signed-rank test.

sBP, systolic blood pressure; dBP, diastolic blood pressure; HR, heart rate. PG, plasma glucose; GH, growth hormone; ACTH, adrenocorticotropic hormone.

^†^sBP, dBP, HR and RHI values at 120 min after insulin administration; PG, adrenaline, noradrenaline and dopamine values at hypoglycemia; ACTH, cortisol and GH values at peak during insulin tolerance test.

### Correlation analysis of factors associated with changes in RHI on insulin tolerance test

**Figure 3:**
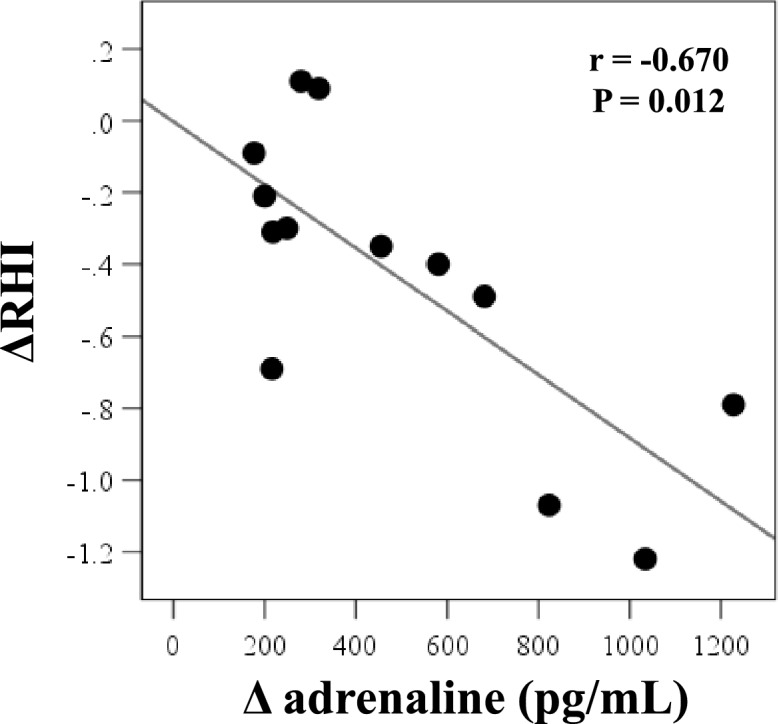
Correlation analysis between changes in RHI and changes in adrenaline during the insulin tolerance test (n = 13). RHI, reactive hyperemia index, ΔRHI, difference between values at baseline and at 120 min after insulin administration; Δadrenaline, difference between values at baseline and at hypoglycemia. ΔRHI, difference between values at baseline and at 120 min after insulin administration; Δadrenaline, difference between values at baseline and at hypoglycemia.

Table 3 shows the correlation between changes in RHI (ΔRHI) and changes in various ITT parameters. ΔRHI correlated inversely and significantly with the changes in adrenaline (Δadrenaline) (r = − 0.670, *p* = 0.012), but not with ΔsBP, ΔdBP, Δpulse rate, Δplasma glucose, ΔACTH, Δcortisol, and ΔGH levels (Fig. 3).

**Table 3:** Correlation analysis of the factors associated with ΔRHI.

	ΔRHI	
	r	*P* value
ΔPG	− 0.079	0.695
ΔsBP	0.096	0.634
ΔdBP	− 0.033	0.871
ΔHR	− 0.265	0.181
ΔCortisol	− 0.084	0.678
ΔACTH	− 0.156	0.788
ΔGH	− 0.054	0.788
Δadrenaline	− 0.670	0.012
Δnoradrenaline	− 0.385	0.194
Δdopamine	− 0.157	0.593

Data are results of Spearman’s correlation analysis.

RHI, hyperemia peripheral arterial tonometry index; sBP, systolic blood pressure; dBP: diastolic blood pressure; HR, heart rate; PG, plasma glucose; ACTH, adrenocorticotropic hormone, GH, growth hormone.

Sampling times for values during insulin tolerance test are listed in Table 2.

## Discussion

Our results showed a decrease in vascular endothelial function (measured by RH-PAT) in association with hypoglycemia in patients free of abnormal glucose tolerance. The decrease in vascular endothelial function correlated with increases in catecholamine levels at the time of hypoglycemia. To our knowledge, there are no reports that have examined the effects of hypoglycemia on vascular endothelial function using RH-PAT in non-diabetic patients. Thus, this study is the first to show the relationship between decreased vascular endothelial function and increased catecholamine levels at the time of hypoglycemia.

Hypoglycemia induces sympathetic nervous system response, altered t-wave morphology, increased pro-coagulant state, inflammation, pro-atheromatous responses, and endothelial dysfunction^22^. Wang et al.^23^ reported that exposure of vascular endothelial cells to hypoglycemia at < 60 mg/dL induced marked decrease in nitric oxide (NO) production, together with marked increase in the production of reactive oxygen species by the mitochondria. These findings suggest that hypoglycemic stimulation increases active oxygen in healthy individuals, together with catecholamine^24^. In this regard, Jin et al.^11^ showed that hypoglycemia-related increase in adrenaline increases the adhesion of monocytes to the vascular endothelium in rats and that excessive catecholamine secretion associated with hypoglycemia leads to vascular endothelial dysfunction. It has also been shown that hypoglycemia increases adrenaline and promotes neointimal formation and smooth muscle cell proliferation via α1 adrenergic receptors after vascular injury^25^. Adrenaline also increases inflammatory cytokines^24,26,27^, which are known to contribute to vascular endothelial dysfunction. However, few studies have reported the effects of hypoglycemic stimulation on vascular endothelial function in non-diabetic patients. Joy et al.^15^ used brachial flow-mediated dilation (FMD) in non-diabetic patients to show that hypoglycemic stimulation increased plasminogen activator inhibitor 1 (PAI-1), vascular cell adhesion molecule-1 (VCAM-1), intracellular adhesion molecule-1 (ICAM-1), E-selectin, P-selectin, thrombin/antithrombin complex, tumor necrosis factor-α, and interleukin-6 responses and reduced endogenous nitric oxide (NO)-mediated vasodilation. The same group reported that hypoglycemic stimulation increased epinephrine and norepinephrine levels in both non-diabetic patients and type 2 diabetic patients, whereas nitroglycerin-mediated exogenous NO-mediated vasodilation decreased only in patients with type 2 diabetes, which is believed to be the mechanism by which hypoglycemia impairs endothelial function^16^.

Our results showed that hypoglycemic stimulation and the associated vascular endothelial dysfunction was at least in part mediated by increased adrenaline levels. To our knowledge, there are no clinical studies that have demonstrated the association of activation of the sympathetic nervous system with hypoglycemia-related decrease in vascular endothelial function. Hypoglycemia activates the hypothalamic–pituitary–adrenal (HPA) axis, and the release of HPA axis hormones has been reported to adversely affect vascular function^28^. Interestingly, in the above study, there was no association between decreased vascular endothelial function and increased cortisol, not even when patients with adrenocortical insufficiency were excluded. These results suggest a larger role for the sympathetic nervous system in the observed decrease in vascular endothelial function than the HPA axis during hypoglycemia. Interestingly, hypoglycemic stimulation is known to increase other insulin counter-regulatory hormones, such as GH, but the increases in these hormones were not associated with vascular endothelial dysfunction.

In a large-scale study of blood glucose control and complications in patients with type 2 diabetes mellitus, it was reported that hypoglycemia correlates significantly with severe cardiovascular adverse events in a group of patients on aggressive glucose-lowering therapy compared with those on standard therapy^29–31^. However, the results of studies on diabetic patients sometimes vary due to a variety of factors, such as duration of diabetes mellitus, diabetic vascular complications, hyperglycemia, fluctuations in blood glucose level, and use of different glucose-lowering agents. On the other hand, our study included only subjects with normal glucose tolerance to elucidate the effects of hypoglycemia on vascular endothelial dysfunction and explore the pathophysiological mechanisms of such effects, without interference by other factors. Since hypoglycemia is known to correlate with vascular endothelial dysfunction in the clinical setting in diabetic patients under treatment, we recommend that diabetic patients who require frequent insulin injections or sulfonylureas (SU) should be provided with a treatment/management protocol that guards against the development of hypoglycemia in order to prevent cardiovascular events.

This study has several limitations. First, the sample size was relatively small, and the subjects were not normal healthy individuals; however, since it is practically impossible to conduct ITT in healthy subjects, only patients who clinically required ITT due to suspected hypopituitarism or hypoadrenocorticism were included to provide the best possible control group. This study was conducted in non-diabetic patients with normal HbA1c and FPG levels; and therefore we expect that the results are similar to those of healthy individuals. Second, there was also no saline control group, thus limiting further comparisons. Third, oxidative stress, inflammatory cytokines, and nitric oxide levels were not evaluated. Finally, insulin itself could have considerable effect on endothelial function, but it was not evaluated. The above issues should be included in future multicenter studies of larger sample size.

In conclusion, our study demonstrated that hypoglycemic stimulation altered vascular endothelial function, as measured by RH-PAT, even in subjects without glucose intolerance. The mechanism of hypoglycemic-induced alteration in vascular endothelial function was related to the associated increase in catecholamine levels at the time of hypoglycemia. Since hypoglycemia seems to be an important factor contributing to the development of vascular endothelial dysfunction, treatment of diabetes mellitus should be tailored to minimize hypoglycemia in order to prevent future cardiovascular events in patients with diabetes mellitus.

## Methods

This single-center prospective observational study was conducted at The University of Occupational and Environmental Health Hospital between June 2017 and April 2019. The study was approved by the ethics review board of the University of Occupational and Environmental Health (approval * UOEHCRB21-061). After selecting the subjects, the study was explained in detail and a signed informed consent form was obtained from each subject in accordance with the Declaration of Helsinki. This study was registered in the UMIN Clinical Trials Registry (UMIN000033244).

### Subjects

Among patients who were hospitalized with suspected endocrine diseases, those who met all the following criteria were included in the study: (1) hospitalized for the diagnosis of hypoadrenocorticism or hypopituitarism; (2) required an insulin tolerance test (ITT) for diagnosis; (3) under 65 years of age at the time the informed consent was obtained; (4) written consent was provided to participate in the study; (5) no abnormal glucose metabolism based on the results of normal HbA1c and FPG levels; and (6) normal vascular endothelial function based on RHI value of ≥ 1.67. Patients who had history of ischemic heart disease, arrhythmia, or epilepsy, or deemed clinically not suitable by the investigators, were excluded.

### Study design

The study protocol is illustrated in Fig. 1. After an overnight fast, the patients were placed on bed rest starting in the early morning. ITT was conducted by measuring plasma glucose and anterior pituitary hormone levels before insulin administration as well as every 30 min until 120 min following the administration. Hypoglycemic stimulation was defined as venous plasma glucose level of ≤ 50 mg/dL after insulin administration. Plasma glucose, anterior pituitary hormone, adrenaline, noradrenaline, and dopamine were also measured at the time of hypoglycemia. Vascular endothelial function was evaluated using a peripheral arterial tonometry (PAT) device (EndoPAT 2000, Itamar Medical, Caesarea, Israel) before and 120 min after insulin administration.

### Insulin tolerance test

After an overnight fast, the patient was placed on bed rest upon reporting to the laboratory at early morning. To induce hypoglycemia, 0.1 unit/kg of insulin was injected intravenously, and the pituitary hormone level was measured every 30 min up to at least 120 min. During ITT, a finger stick blood test was performed using Medisafe FIT^Ⓡ^ (Terumo Co., Tokyo, Japan) every 15 min. If severe hypoglycemic effect was observed (clinically manifested by convulsions or loss of consciousness), testing was terminated immediately, and 20 mL of 20% dextrose was rapidly injected intravenously. If hypoglycemia (blood glucose level < 70 mg/dL) persisted at test completion, 20 mL of 20% dextrose was injected rapidly intravenously.

### Noninvasive vascular function test

The test was performed in a temperature-controlled room (21–24 °C) after 30-min rest in supine position. The baseline pulse amplitude was recorded during a 5-min period before the induction of local ischemia. Ischemia was induced by placing the blood pressure cuff on the upper arm, while the opposite arm served as a control. The peripheral arterial tonometry probes were placed on one finger of each hand. After 5 min, the blood pressure cuff was inflated to 60 mmHg above the systolic pressure or to 200 mmHg for 5 min and then deflated to induce reactive hyperemia. As a measure of reactive hyperemia, RHI was calculated as the ratio of the average amplitude of the peripheral arterial tonometry signal over 1 min beginning 1.5 min after cuff deflation (control arm, A; occluded arm, C) divided by the average amplitude of the peripheral arterial tonometry signal over the 2.5-min time period before cuff inflation (baseline) (control arm, B; occluded arm, D). Thus, RHI = (C/D)/(A/B) × baseline correction. Because RHI has a heteroscedastic error structure, we used the natural logarithm transformation in all analyses. Vascular endothelial dysfunction was defined as RHI < 1.67^32,33^.

### Blood tests

HbA1c levels (%) were measured using a high-performance liquid chromatography method with a Tosoh HLC-723 G8 analyzer (Tosoh Co., Kyoto, Japan) and expressed in National Glycohemoglobin Standardization Program (NGSP) equivalent values calculated from the following equation: HbA1c (NGSP) = HbA1c (Japan Diabetes Society [JDS]) (%) + 0.4%^34^. ACTH and cortisol levels were measured using electro chemiluminescence immunoassay (the Hospital of the University of Occupational and Environmental Health, Japan, Kitakyushu, Japan). Growth hormone (GH) levels were measured using electro chemiluminescence immunoassay (SRL Co., Tokyo, Japan). IGF-1 levels were measured using electro chemiluminescence immunoassay (LSI Medience Co., Tokyo, Japan). Adrenaline, noradrenaline and dopamine levels were measured using high-performance liquid chromatography (SRL Co., Tokyo, Japan).

### End points

The primary endpoint was a change in vascular endothelial function 120 min after insulin administration. The secondary endpoints were factors related to changes in vascular endothelial function.

### Statistical analysis

Data were expressed as mean ± standard deviation. The Wilcoxon signed-rank test was used to compare parameters before and after insulin administration. Spearman's correlation analysis was used to correlate changes in RHI with changes in each parameter evaluated. Statistical significance was set at *P* value of < 0.05. All statistical analyses were performed using SPSS version 25.0 (SPSS Inc., Chicago, IL).

## Data Availability

All data generated or analyzed during this study are included in the published article.
